# Invasive pulmonary aspergillosis with no abnormal immunity overlapped with allergic bronchopulmonary aspergillosis: A case report

**DOI:** 10.1097/MD.0000000000046399

**Published:** 2026-01-02

**Authors:** Jueqi Lv, Qian Ning, Chunxing Que, Yanbin Wu

**Affiliations:** a Respiratory and Critical Care Medicine, The First Affiliated Hospital of Guangxi Medical University, Nanning, China.

**Keywords:** ABPA, Aspergillus, Aspergillus overlap syndrome, IPA

## Abstract

**Rationale:**

Invasive pulmonary aspergillosis (IPA) overlapping with allergic bronchopulmonary aspergillosis (ABPA) is an exceedingly rare disease called *Aspergillus* overlap syndrome. Fewer than 30 cases have been reported in the literature since 1936. Furthermore, early diagnosis and treatment improve the prognosis of patients, especially those without underlying diseases.

**Patient concerns:**

A 45-year-old male patient with no underlying disease who was admitted to the hospital with cough, expectoration, fever, and shortness of breath. The patient was diagnosed with pulmonary aspergillosis overlap syndrome, which progressed from IPA to ABPA.

**Diagnoses:**

IPA overlapping with ABPA.

**Interventions:**

Early diagnosis and timely anti-Aspergillus and steroid treatment.

**Outcomes:**

The patient’s symptoms improved, and the pulmonary lesions gradually resolved.

**Lessons:**

This study emphasizes that patients with IPA without underlying diseases should be alert to ABPA overlap, and early combination therapy can improve prognosis.

## 1. Introduction

Invasive aspergillosis is a severe disease, particularly in immunocompromised individuals, with a high mortality rate. It often occurs in those with conditions like chronic obstructive pulmonary disease, intensive care unit admission, lung cancer, or hematologic malignancy.^[1]^ The spectrum of pulmonary aspergillosis includes allergic bronchopulmonary aspergillosis (ABPA), chronic pulmonary aspergillosis, and invasive pulmonary aspergillosis (IPA), distinguished by immune status and disease risk.^[2–4]^ ABPA usually affects patients with preexisting allergic lung diseases, while IPA is more prevalent in immunocompromised hosts. Although there is evidence that patients with ABPA may develop IPA due to immunosuppression due to long-term glucocorticoid use, progression from IPA or concurrent ABPA in patients without underlying disease has not been well documented. This paper reports a case of IPA overlapping with ABPA.

## 2. Case presentation

A 45-year-old man, a vegetable farmer living in southern China, presented to the emergency department with a history of cough, expectoration, fever for >1 month, and shortness of breath for 13 days. He had a history of smoking and alcohol consumption for over 20 years but no history of allergies or asthma.

**Figure 1.**
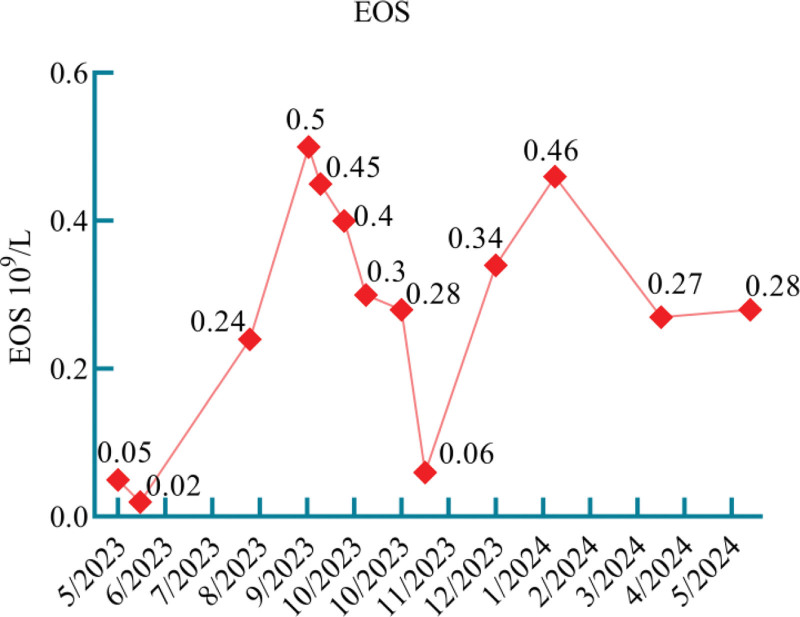
Figure shows the number of EOS in the peripheral blood of the patient, which is 0.5 × 10^9^/L only once, and <0.5 × 10^9^/L in the rest. EOS = eosinophils.

**Figure 2.**
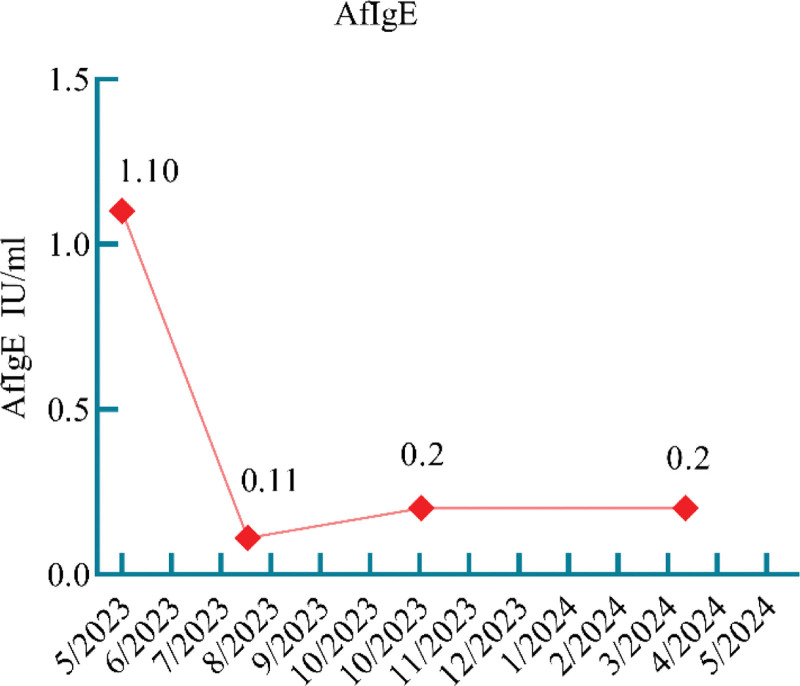
Figure shows the patient’s serum *Aspergillus fumigatus* IgE (AfIgE, negative < 0.35 IU/mL, positive ≥ 0.35 IU/mL), it can be seen that the initial treatment was positive, and the treatment and follow-up were negative. AfIgE = *Aspergillus fumigatus* immunoglobulin E.

**Figure 3.**
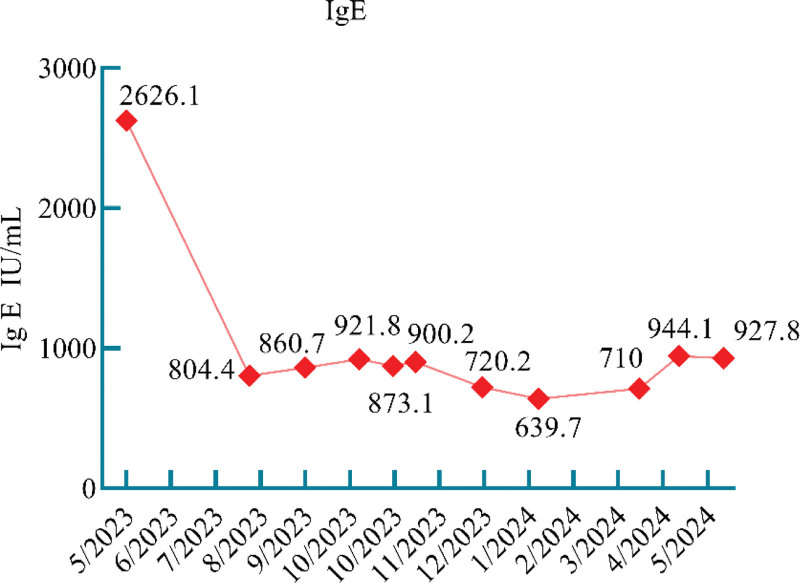
Figure shows total serum IgE, normal range 1 to 100, and one of the diagnostic criteria for ABPA is IgE > 500 IU/mL. ABPA = allergic bronchopulmonary aspergillosis, IgE = immunoglobulin E.

One month before admission to our hospital (on April 5, 2023), the patient developed a cough, expectoration, fever, white viscous sputum, and a temperature of up to 39.9°C, accompanied by shortness of breath after exercise. On May 2, 2023, the patient’s shortness of breath worsened, accompanied by wheezing, being unable to walk, and being unable to lie flat at night. And he was hospitalized at another hospital. The main laboratory examination in the other hospital showed that Aspergillus fumigates had been cultured in bronchoalveolar lavage fluid (BALF) several times, and the *G* test (BALF) was 669.53 pg/mL. Chest computerized tomography (CT) showed bilateral lung infection and bilateral hilar and mediastinal lymph enlargement, accompanied by a small amount of right pleural effusion. IPA was diagnosed in the other hospital and treated with intravenous voriconazole (6 mg/kg once every 12 hours in the first 24 hours, 4 mg/kg once every 12 hours thereafter; from May 3 to 8), anti-Aspergillus treatment. During the period, the patient developed delirium, such as irritability and gibberish, and an adverse drug reaction to voriconazole was considered. The blood concentration of voriconazole was 9.14 ng/mL (0.5–5 µg/mL). Voriconazole was discontinued, and the patient was shifted to antifungal therapy combining caspofungin (70 mg on day 1 and 50 mg once daily later; May 5–15) and amphotericin B (gradually increasing the dose to 40 mg/d; May 8–15), supplemented with high-flow oxygen therapy (flow rate 40–50 L/min, oxygen concentration 60–90%). However, the patient still had a fever, cough, phlegm, shortness of breath, thick yellow phlegm, sometimes bloody sputum, fatigue, and a poor appetite. The breath sounds were coarse in both lungs, and numerous moist rales and scattered wheezing were heard. Bronchoscopy in the other hospital showed congestion, edema, and leukoplakia of the bronchial mucosa. Reexamination of the lung CT showed that the lesions in both lungs progressed and the cavity size increased. On May 15, 2023, he was thus transferred to our respiratory intensive care unit. Here, his laboratory results suggested IPA and ABPA (Figs. 1–3). Skin prick tests for Aspergillus were not performed because they were not provided at our center.

**Figure 4.**
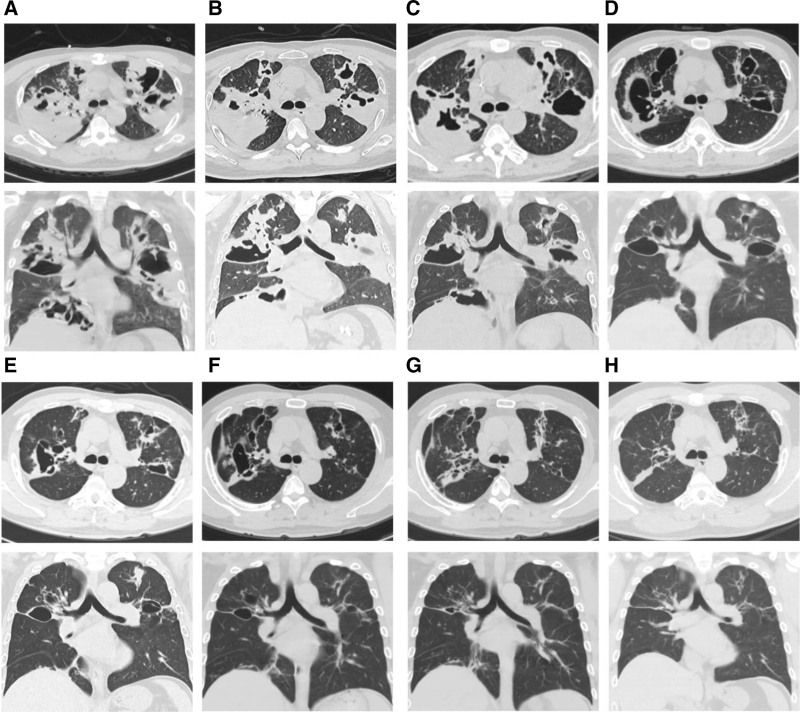
Figure shows dynamic observation of lung CT. (A) Chest CT upon admission to our hospital (May 15, 2023) shows multiple patchy opacities, consolidations, and cavitary lesions in both lungs. (B) One week after combination therapy (May 22, 2023), some lesions show slight absorption. (C) At 2 wk (May 29, 2023), there is a reduction in consolidation and cavity size. (D) At 2 mo (July 2023), further resolution of lesions is observed. (E) At 3 mo (August 2023), continued improvement with significant shrinkage of cavities. (F) At 5 mo (October 2023), lesions are markedly absorbed. (G) At 6 mo (November 2023), only minimal residual streaks and nodules remain. (H) At 1 yr (May 2024), the pulmonary lesions have almost completely resolved. CT = computerized tomography.

According to the EORTC-MSGERC criteria for IPA, the patient’s clinical features were consistent with refractory fever, dyspnea, and cavitation on chest CT (Fig. 4). The fungal etiology was consistent with a positive serum Aspergillus fumigate-specific antibody, 2 consecutive serum GM positives, and BALF next generation sequencing suggestive of Aspergillus (Table 2). Based on the characteristics of the appeal, we diagnosed it as IPA. According to the International Society for Human and Animal Mycology (ISHAM) criteria for ABPA diagnosis (Table 1), 2 major and 2 minor criteria were met, including (major criteria) an elevated serum total immunoglobulin E (IgE) level and an elevated Aspergillus-specific IgE, and (minor criteria) Aspergillus serum immunoglobulin G antibodies (Table 2) and a chest CT suggesting tracheal dilatation.

**Table 1:** Diagnostic criteria for ABPA (ISHAM 2013) and comparison with the present case.

Category	Diagnostic criteria (ISHAM 2013)	Present case
Prerequisite condition	Asthma or cystic fibrosis	No asthma or cystic fibrosis
Obligatory criteria (both required)	(1) Type I hypersensitivity to *Aspergillus fumigatus* (positive skin test or elevated specific IgE) (2) Serum total IgE > 1000 IU/mL (A level < 1000 IU/mL may be acceptable if all other criteria are fulfilled)	(1) Elevated specific IgE (2) Total serum IgE > 1000 IU/mL
Supportive criteria (≥2 required)	(1) Elevated *Aspergillus fumigatus*–specific IgG (or precipitating antibodies) (2) Radiologic findings consistent with ABPA* (3) Peripheral blood eosinophilia > 0.5 × 10^9^/L^†^	(1) Elevated specific IgG (2) CT suggesting bronchiectasis (3) Eosinophilia > 0.5 × 10^9^/L (once)
Diagnostic conclusion	Meets prerequisite, both obligatory, and ≥2 supportive criteria → ABPA	Met criteria for ABPA^‡^ despite no asthma history

ABPA = allergic bronchopulmonary aspergillosis, CT = computed tomography, Ig = immunoglobulin, ISHAM = International Society for Human and Animal Mycology.

* Pulmonary opacities may be transient (e.g., fleeting opacities, consolidation, finger-in-glove sign) or fixed (e.g., central bronchiectasis).

† Eosinophilia may be a historical finding.

‡ According to ISHAM criteria, ABPA can be considered in patients without asthma if all other criteria are fulfilled. Skin prick test was not performed in this case.

**Table 2:** Laboratory results of the patient at the initial visit.

Inspection data	Value	Reference range
Serum IgE	2626.1	0–100
Serum IgE antibody against *Aspergillus fumigatus*	1.10	Negative < 0.35 IU/mL Positive ≥ 0.35 IU/mL
Serum IgG antibody against Aspergillus SPP	300.86	Negative < 80.00 Positive > 120.00 Grey zone 80~120
Serum Aspergillus galactomannan antigen	1.705	Negative < 0.5 Positive > 0.5
Serum fungal (1–3) -β-d-glucans	698.18	0–100
BALF fungal (1–3) -β-d-glucans	980.65	0–100
BALF Aspergillus galactomannan antigen	4.487	Na
BALF mNGS	*Aspergillus fumigatus*	Na
BALF fungal fluorescence examination	Hyphae and spores	Na
BALF Aspergillus galactomannan antigen	6.287	Na

BALF = bronchoalveolar lavage fluid, IgE = immunoglobulin E, IgG = immunoglobulin G, mNGS = metagenomic sequencing of bronchoalveolar lavage fluid.

**Figure 5.**
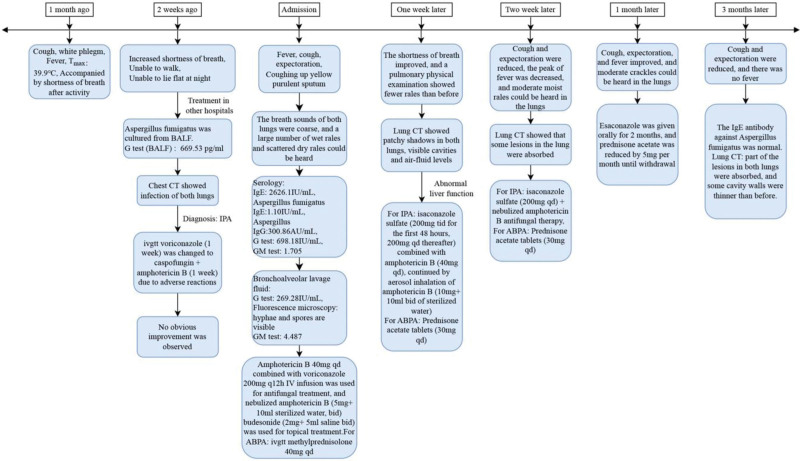
Figure shows progress in diagnosis and treatment. This flowchart illustrates clinical events from initial symptom onset to follow-up. *G* test: (1,3)-β-d-polyglucosan test. GM test: galactomannan test, qd: once daily, bid: twice daily, tid: 3 times daily, CT = computerized tomography, BALF = bronchoalveolar lavage fluid, IgE = immunoglobulin E, IgG = immunoglobulin G.

We considered that the patient presented with IPA development in combination with ABPA. For IPA, we used antifungal therapy with amphotericin B 40 mg once daily combined with voriconazole 200 mg once every 12 hours IV infusion and topical treatment with nebulized amphotericin B (5 mg + 10 mL sterilized water, twice daily) and budesonide (2 mg + 5 mL saline, twice daily). For ABPA, we used intravenous methylprednisolone 40 mg once daily for anti-inflammatory. One week later (May 22, 2023), the patient’s shortness of breath improved, and a pulmonary physical examination showed that the dry and wet rales were less than before. Unfortunately, the patient developed liver dysfunction, which was considered to be related to the drug, and the antifungal medication was changed to isaconazole sulfate (200 mg 3 times daily for the first 48 hours and 200 mg once daily thereafter) combined with amphotericin B (40 mg once daily), followed by aerosol inhalation of amphotericin B (10 mg + 10 mL twice daily of sterilized water). Intravenous methylprednisolone was discontinued and shifted to prednison acetate tablets (30 mg once daily) to continue treatment. After treatment, the patient’s cough and expectoration were reduced, the peak value of fever decreased, and a moderate amount of wet rales could be heard in the lungs without dry rales. Intravenous amphotericin B was discontinued after 2 weeks, and antifungal therapy with isaconazole sulfate (200 mg once daily) and nebulized amphotericin B was continued. Intravenous esaconazole sulfate was used for 1 month and then changed to oral administration for another 2 months. Prednisone acetate tablets were gradually tapered. During the diagnosis and treatment process, eosinophilia in peripheral blood > 0.5 × 10^9^/L once (Fig. 1). Aspergillus fumigate IgE antibodies had decreased to the normal range by 3 months after treatment (Fig. 2). The patient had been followed up regularly since discharge. Reexamination of IgE showed a downward trend (Fig. 3), but it did not fall to the normal range. Dynamic observation of lung CT (128-slice) plain scan showed that the lung lesions gradually improved (Fig. 4). After treatment and close monitoring, the patient’s symptoms gradually improved. Respiratory symptoms decreased, peak fever decreased, and physical examination of the lungs improved. The course of the patient’s diagnosis and treatment is shown in Figure 5.

The pulmonary lesions showed gradual shrinkage and improvement during the follow-up period. After half a year of treatment, the patient had no symptoms and returned to normal life.

## 3. Discussion

This case report reveals a rare clinical phenomenon: the development of an overlap syndrome of ABPA in a patient with IPA without underlying disease. Pulmonary aspergillosis encompasses a spectrum of diseases, including ABPA, chronic pulmonary aspergillosis, and IPA, each with distinct clinical presentations and host immune statuses. ABPA typically affects individuals with preexisting allergic lung diseases such as asthma, while IPA is more prevalent in immunocompromised hosts. The overlap of these conditions, known as Aspergillus overlap syndrome, is rare but has been increasingly recognized. In this case, the patient developed IPA overlapping with ABPA despite the absence of underlying disease or immunosuppression, suggesting that specific environmental exposures, such as prolonged contact with Aspergillus spores, can trigger such infections. In this case, the patient had neither underlying disease nor immunosuppression, but was diagnosed with IPA overlapping with ABPA, which is worth learning.

Early diagnosis of IPA is crucial for improving patient outcomes. While histopathological examination is considered the gold standard,^[5]^ it is not always feasible. Metagenomic sequencing (mNGS) of BALF and CT imaging have emerged as valuable tools for early detection. It has been proposed that mNGS provides a feasible and very sensitive method for detecting Aspergillus in immunocompromised patients, and that the ability of mNGS to accurately identify fungal species and co-pathogens helps guide appropriate antimicrobial therapy.^[6]^ In addition, lung CT also plays an important role in the early diagnosis of IPA. The pathogenesis of IPA can be categorized into vascular invasion and airway invasion patterns.^[7]^ The former is common in patients with severe neutropenia and is characterized by fungal thrombosis and necrosis,^[8,9]^ while the latter is seen in non-neutropenic patients and involves extensive endobronchial infection.^[7,8]^ The imaging features of IPA vary depending on the pattern and stage of infection, with early CT scans showing small nodules and a halo sign, which may evolve into cavities as the disease progresses. These 2 patterns can be intertwined in the same patient,^[7]^ and some patients only show consolidation or ground glass opacity.^[10,11]^

We observed that patient had no history of asthma, but patient final diagnosis of IPA overlapped with ABPA. According to the criteria of the ISHAM ABPA Working Group, a diagnosis of ABPA can be considered in patients who meet other criteria, even if there is no history of asthma. This highlights the need to consider in the process of diagnosis of a wide range of clinical and laboratory parameters. Typically, the diagnosis of ABPA requires the presence of asthma or another underlying medical condition. However, a retrospective analysis found that about 7% of 530 patients with ABPA did not have concomitant asthma.^[12]^ Nevertheless, a higher incidence of ABPA without asthma has been reported in Japanese patients, about 20%.^[13]^ According to the ISHAM ABPA working group criteria (2013; Table 1),^[14]^ ABPA can be considered if the patient without asthma meets other criteria. However, it needs to be differentiated from allergic bronchial mycosis (ABPM). ABPM is a hypersensitive disease caused mainly by fumigates, but also by Candida albicans, Bipolaris spp, Schizophyllum commune, and other pathogenic bacteria.^[15–17]^ ABPM is currently understudied, and further basic and clinical studies are needed in order to understand its epidemiological characteristics and to design more effective treatments. Compared with ABPA, the prevalence of ABPM caused by other fungi is much lower. The diagnostic criteria of ABPM are similar to those of ABPA, but the differential diagnosis is mainly based on the isolation of pathogenic bacteria and the demonstration of sensitization of the body to the pathogenic fungi.^[18]^

In this case, patients early didn’t use hormones, nor use of itraconazole, no basic diseases, the reason of IPA developed into the overlap of ABPA worthy of our thought. First of all, the occurrence of both IPA and ABPA in the same patient is rare, and it may be a coincidence. Genetic factors may also predispose patients to progress from 1 form of aspergillosis to another. For example, mutations in the CFTR gene may lead to ABPA, and mances-binding lectin gene mutations may result in chronic necrotizing aspergillosis or IPA.^[19]^ Immunosuppression is a major risk factor for the development of IPA.^[20]^ Although the patient in this case did not use corticosteroids or itraconazole, it is still possible that other factors, such as an increased fungal load, could have led to the development of IPA. What`s more, The progression from IPA to ABPA may be related to changes in the host immune status. In ABPA, there is a type 2 immune response characterized by the production of IgE and eosinophilia.^[21]^ It is possible that the immune response to the Aspergillus infection in this patient shifted from a primarily antifungal response to a type 2 immune response, leading to the development of ABPA. Further, the virulence and immunoevasive properties of *Aspergillus fumigatus* may contribute to its ability to cause both invasive and allergic disease.^[4]^ The fungus may have initially caused IPA, but then triggered an allergic response in the patient, leading to the development of ABPA. Now, the exact reason why only a minority of patients with ABPA continue to develop IPA remains a mystery, and it is worth pondering why it is even rarer for IPA to develop into ABPA. In this case, the exact mechanism of the patient’s progression from IPA to ABPA is not clear, and it may be related to environmental exposure, changes in the host immune status, and side effects of drug treatment. Therefore, we cannot conclude that the phenomenon observed in the present case is universal, and more case studies and epidemiological data are needed to support it.

The patient reported in this case was a farmer who often used poultry manure as an organic fertilizer to grow vegetables. He planted a vegetable field next to the accumulation of the poultry excrement and urine, and poultry manure and its surrounding humidity environment may cause a lot of fungi, including Aspergillus spores. Patients may take a long time to come into contact with a lot of Aspergillus fumigate spores, and possibly in a short time, a large number are inhaled through the respiratory tract, leading to infection. And Aspergillus spores are widespread in indoor and outdoor air, sourced from soil, plants, etc.^[22,23]^ Environmental factors like construction and pollution increase the risk of IPA,^[24–30]^ even for immunocompetent adults. It has been reported that immunocompetent adults exposed to high-risk factors may also develop IPA.^[31]^ A questionnaire-based study found that patients with ABPA were more likely to come from rural areas compared to asthmatic patients.^[32]^

In this case, the report is the main lesson of the clinicians in the management of patients with IPA, should be vigilant against the potential ABPA overlap, especially under the condition of the patients with a history of exposure to Aspergillus spores. Early recognition and comprehensive management of the overlap of IPA and ABPA are essential to optimize patient outcomes. In addition, management of environmental factors, such as reducing exposure to Aspergillus spores, may help prevent the development of pulmonary aspergillosis.

As for strengths, we not only provide detailed longitudinal imaging, laboratory, and treatment data, but also application of both EORTC-MSGERC and ISHAM criteria to objectively define IPA and ABPA. Moreover, this is the first report of IPA-to-ABPA progression in an immunocompetent host without underlying disease. However, it also has some limitations. Single-case observational design, limiting generalizability. Absence of genetic or immune-phenotyping analyses to explore mechanisms. Although this case provides valuable insights, whether these specific conclusions can be elevated to universal truths needs to be tested in future research. More extensive epidemiological studies are recommended to determine the incidence of IPA and ABPA overlap, risk factors, and optimal treatment strategies.

## 4. Conclusions

The progression from IPA to concomitant ABPA is a rare and alarming condition. Clinicians need to fully understand the characteristics of the 2 diseases and conduct comprehensive evaluation and diagnosis when dealing with such cases. Clinicians should maintain keen vigilance for the presence of combined ABPA in patients having respiratory symptoms and evidence of IPA, especially in individuals with Aspergillus spore exposure. This case highlights the need for early recognition and comprehensive management of combined IPA and ABPA to optimize patient outcomes and reduce the risk of treatment-related complications.

## Acknowledgments

We would like to express our gratitude to the patient for granting permission to use their clinical data in this paper and for the publication of this research. We are also grateful to all the researchers, including the physicians, nurses, and technicians, who participated in this study.

## Author contributions

**Conceptualization:** Yanbin Wu.

**Data curation:** Jueqi Lv, Qian Ning, Chunxing Que.

**Formal analysis:** Jueqi Lv, Qian Ning, Chunxing Que.

**Funding acquisition:** Yanbin Wu.

**Investigation:** Jueqi Lv.

**Methodology:** Jueqi Lv.

**Project administration:** Jueqi Lv, Yanbin Wu.

**Resources:** Yanbin Wu.

**Writing – original draft:** Jueqi Lv.

**Writing – review & editing:** Yanbin Wu.
